# The role of social network diversity in self-perceptions of aging in later life

**DOI:** 10.1007/s10433-024-00815-z

**Published:** 2024-06-26

**Authors:** Frauke Meyer-Wyk, Susanne Wurm

**Affiliations:** 1 Department for Prevention Research and Social Medicine, Institute for Community Medicine, University Medicine Greifswald, Greifswald, Germany; 2 European Commission, Joint Research Centre (JRC), Ispra, Italy

**Keywords:** Self-perceptions of aging, Views on aging, Social networks, Social roles, Network diversity

## Abstract

While the link between self-perceptions of aging (SPA) and healthy aging is well established, less is known about the association between social factors and SPA. The present study investigated whether higher social network diversity is associated with more positive and less negative SPA and whether this association is moderated by age. We examined cross-sectional data from the German Ageing Survey of 2008 (DEAS; *N* = 6205, 40–85 years, 49.5% female). Network diversity was assessed as the number of social roles in an individual’s network (such as spouse, friend and colleague). Three domains of SPA were measured using the Aging-Related Cognitions Scale (AgeCog): ongoing development (positive SPA), social losses (negative SPA) and physical losses (negative SPA). We conducted multiple linear regression models and tested for a moderator effect of age using an interaction term of age and network diversity. Results showed that at higher ages older adults with higher network diversity reported more positive SPA related to ongoing development and more negative SPA related to social losses than those with less diverse networks, indicating that age has a moderating effect. We found no association between network diversity and negative SPA related to physical losses and no indication that age was relevant to this relationship. The present study adds to evidence on the role of social networks in SPA. Our findings suggest that in certain SPA domains and depending on age, network diversity is related to both more positive and more negative SPA, which emphasizes the importance of considering domain-specific SPA.

**Supplementary Information:**

The online version contains supplementary material available at 10.1007/s10433-024-00815-z.

## Introduction

Aging is a process that is common to all people. What is distinctive, however, are the personal beliefs and experiences that accompany this process. These *self-perceptions of aging* (SPA) are known to be intricately related to individuals’ health (Westerhof et al. 2023). However, comparatively little is known about the association between social factors and SPA. Recent studies have provided initial insights into this relationship and have shown that certain structural characteristics of older adults’ social networks such as network size (Kim et al. 2012; Jung et al. 2021) and age integration (i.e., inclusion of younger people in one’s personal network; Cohn-Schwartz et al. 2022) are related to individuals’ SPA. One factor that has not been considered so far, however, is social network diversity, i.e., the number of different social roles in older adults’ social networks. Using data from the German Ageing Survey (DEAS), the present study examines the role of network diversity in the context of SPA, which are considered important mindsets for successful aging (Sabatini et al. 2024).

### Self-perceptions of aging

SPA refer to an individual’s perceptions of their own aging process and the state of being old (Wurm et al. 2017). SPA constitute the subjective component of the overarching concept *views on aging*. A second component, age stereotypes, is constituted by *societal* concepts of aging and older adults (Wurm et al. 2017). Theoretical assumptions (Levy 2009) and empirical evidence (Kornadt et al. 2017; Rothermund and Brandstädter 2003) suggest that age stereotypes and SPA are closely interrelated and that age stereotypes serve as a substantial basis for the development of individuals’ personal SPA. Given their close relationship, it is common practice to include only one of the two concepts in statistical modeling of studies that are interested in either SPA or age stereotypes as an outcome in relation to other variables, in order to obtain meaningful results regarding the variance explained by those other variables (Kornadt et al. 2017, 2020). The present study follows the same approach and focuses on SPA.

While many previous studies have been based on a unidimensional concept of SPA representing adults’ SPA on a positive–negative continuum, more recent studies have increasingly conceptualized SPA as a multidimensional construct (Diehl et al. 2014; Rothermund and Kornadt 2015; Wurm et al. 2007). When individuals think about their own aging (SPA), they often associate it with both negative and positive attributes (Heckhausen et al. 1989; Hummert 2011; Keller et al. 1989). Negative sides of aging often relate to physical losses (e.g., loss of energy, health problems), or social losses (e.g., loss of a partner or of social roles, feelings of loneliness; Steverink et al. 2001), while the positive sides of aging may be perceived to be ongoing personal development, such as having more freedom and time to pursue plans (Steverink et al. 2001), and gaining a sense of maturity (Heckhausen et al. 1989). Both cross-sectional age group comparisons (Beyer et al. 2017) and longitudinal age trajectories (Diehl et al. 2021) have shown that multidimensional SPA vary according to age. Specifically, Diehl et al.’s (2021) study of adults aged between 40 and 85 showed an increase in the perception of physical and social losses from the age of 65, while perceptions of ongoing development started declining from the age of 55, and displayed a steeper decline from the age of 70. These results suggest that a multidimensional concept of SPA better reflects the fact that individuals perceive aging differentially in different life domains (Baltes 1987). The present study follows this multidimensional approach and focuses on the three SPA domains commonly termed SPA ongoing development, SPA social losses and SPA physical losses (Wurm et al. 2007; Kornadt et al. 2020).

### Social network diversity in later life

Social networks can be defined as a “set of actors and the ties among them” (Wasserman and Faust 1994, p. 9). One type of tie is the social role an actor has vis-à-vis another, such as “kin” or “friend” (Borgatti et al. 2009). The number of different social roles represented within an individual’s personal network is referred to as network diversity (Cohen et al. 1997; Ellwardt et al. 2015; Ali et al. 2018), which describes the structure of a social network. Several gerontological theories have dealt with social network structure in older age, and especially with changes in network structure over the life course (e.g., Socioemotional Selectivity Theory, Carstensen 1992; Convoy Model; Antonucci et al. 2014). Yet, to the best of the present authors’ knowledge, only one theoretical model has put forward explicit assumptions about the effects of network diversity on older adults. The Differential Investment of Resources (DIRe) model (Huxhold et al. 2022) posits an interrelationship between an individual’s personal network and the context and personal characteristics of the individual. More specifically, the model assumes that a social network fulfills specific social functions for the individual and that diverse social networks offer the most beneficial combination of social functions to best cope with age-related changes.

Diverse social contacts were found to be associated with more diverse behavior (Fingerman et al. 2020) and with better cognitive functioning (Ali et al. 2018) among older adults. These findings suggest that network diversity has a stimulating effect, which in turn gives rise to the assumption that greater network diversity could be related to more positive SPA ongoing development. Furthermore, older adults in diverse social networks were found to be less likely to feel lonely than older adults in restricted social networks (Litwin and Shiovitz-Ezra 2011), suggesting that individuals’ social evaluation of their personal network is more positive in diverse networks. Accordingly, greater network diversity could also be associated with less negative SPA social losses. Finally, studies have shown that network diversity is related to more physical activity (Litwin 2003; Fingerman et al. 2020) and to better physical functioning (Ali et al. 2018) among older adults. Against this background, it is likely that greater network diversity is linked to less negative SPA physical losses.

In line with other theories (Carstensen 1992; Antonucci et al. 2014), the DIRe model assumes that social networks generally tend to become smaller and less diverse in older age groups due both to individual factors such as declining health and contextual factors such as retirement (Huxhold et al. 2022). As partial support for these assumptions, several studies have shown a reduction in network size in older age (Wrzus et al. 2013; Cornwell et al. 2008; English and Carstensen 2014), with fewer extrafamilial and peripheral contacts (Wrzus et al. 2013; Cornwell et al. 2008; English and Carstensen 2014; van Tilburg 1998; Barnes et al. 2004; Conway et al. 2013). The level of kinship and close ties in older age, on the other hand, was found to remain stable (English and Carstensen 2014; Shaw et al. 2007) or even to increase (van Tilburg 1998; Litwin et al. 2020). However, it is noteworthy that the DIRe model also recognizes that certain individual and contextual factors may prevent older adults from transitioning to smaller, more homogeneous networks. Some studies support this notion by showing that networks either remain at a stable size with increase in age (van Tilburg 1998), or even expand and transform by the addition of new members (Schwartz and Litwin 2018). Nevertheless, the majority of research suggests that network diversity diminishes and the prevalence of certain social roles changes across age groups. Against this background, and given the DIRe model's assumption that network diversity is beneficial in compensating for age-related changes, it is likely that network diversity is of greater relevance for SPA in older adults than in middle-aged adults.

### The role of social networks in SPA

Some recent findings exist on the relationship between social networks and SPA, but none concerning network diversity. However, certain studies have looked at related structural network characteristics. Network size, for instance, which is a logical prerequisite for network diversity, has been found to be associated with more positive SPA in general (Kim et al. 2012) and specifically more positive SPA ongoing development (Jung et al. 2021). Conversely, smaller social networks (Jung et al. 2021) and social isolation (Santini et al. 2019) are related to more negative SPA. In addition, two recent studies have looked at the importance of the age composition of personal networks in SPA. One of these studies suggested that older adults whose social network included members more than 10 years younger than them held more positive SPA (Cohn-Schwartz et al. 2022), whereas the other study suggested that contact with other older adults, in particular in the domains of friends and leisure, is also important in older adults’ SPA (Cohn-Schwartz et al. 2023). Although these studies focused on age diversity rather than network diversity per se, the findings raise the question of whether greater network diversity may be associated with more positive SPA in later life. The state of research does not yield clear conclusions. However, it provides tentative indications for the present study, suggesting that there is a positive relationship between larger and more age-diverse networks and more positive SPA among older adults.

## The present study

The present study aims to further our understanding of the association between social factors and SPA. Previous research suggests that certain structural characteristics such as network size and age composition play a role in SPA, and this points to network diversity also potentially being relevant. However, a link of this nature has not yet been researched. To address this gap, the present study investigates the relationship between network diversity and SPA in older adults and examines assumptions about this relationship derived from the DIRe model (Huxhold et al. 2022). We base our hypotheses on the DIRe model’s premise that diverse social networks provide the most favorable combination of social functions for coping with age-related changes, as well as on empirical findings that show a positive relationship between network diversity and indicators of cognitive health and active aging, of socio-emotional well-being and of physical health among older adults (Fingerman et al. 2020; Ali et al. 2018; Litwin 2003; Litwin and Shiovitz-Ezra 2011). Taken together, these lead us to assume that network diversity may play a beneficial role in individuals’ perception of their own aging in the domains of ongoing development, physical losses and social losses.

Accordingly, we propose the following hypotheses:

### H1

Greater network diversity is associated with more positive SPA related to ongoing development.

### H2

Greater network diversity is associated with less negative SPA related to social losses.

### H3

Greater network diversity is associated with less negative SPA related to physical losses.

Considering the findings that both network diversity and SPA differ with age, we further assume that chronological age may have a moderating effect which should be taken into account in the relationship between network diversity and SPA, since maintaining a high level of network diversity may particularly contribute to more positive, or less negative SPA in older age. Accordingly, we hypothesize:

### H4

The strength of the relationship between network diversity and SPA is moderated by chronological age, i.e., the relationship becomes more pronounced in older age.

## Methods

### Sample

Data was derived from the German Ageing Survey (DEAS), a representative, register-based, large-scale study of community-dwelling adults aged 40 and older in Germany. The present study used a cross-sectional sample of 6205 individuals recruited for the first time in 2008. The response rate, i.e., the number of valid interviews as a proportion of the gross sample of eligible people, amounted to 35.7% in 2008. Data was collected by means of a computer-assisted personal interview and a self-report questionnaire (“drop-off”) (Klaus et al. 2017).

### Measures

#### Self-perceptions of aging (SPA)

SPA were assessed using the Aging-Related Cognitions Scale (AgeCog; Steverink et al. 2001; Wurm et al. 2007). The scale includes three subscales, each reflecting one of the three SPA domains physical losses, social losses and ongoing development (Steverink et al. 2001; Wurm et al. 2007). The first subscale refers to the perception of aging as being accompanied by physical losses. One example item is “Aging means to me that I cannot make up for my physical losses.” The second subscale, social losses, refers to whether aging is perceived as being accompanied by loss of social status. It includes items such as: “Aging means to me that I feel less respected.” The third subscale refers to the perception of aging as a phase of ongoing personal development and contains items such as “Aging means to me that I can still learn new things.” Each subscale consists of four items with a 4-point Likert-scale from 1 (Strongly agree) to 4 (Strongly disagree). Calculation of Cronbach's alpha revealed good reliability for each subscale (SPA physical losses: Cronbach’s *α* = 0.78; SPA social losses: Cronbach’s *α* = 0.76; SPA ongoing development: Cronbach’s *α* = 0.82). We reverse coded the scores and then averaged them for each subscale. The higher the score the stronger the respondent’s perception of physical losses, social losses and ongoing development, respectively.

#### Social network diversity

Participants were asked to report up to eight people whom they considered important and with whom they had regular contact. They were then asked to provide additional information on each person mentioned. One such item referred to the social role of the respective person in the participant’s life and asked “What is your relationship to this person?” Forty-nine categories were presented to choose from (e.g., spouse/partner, child, friend, colleague, neighbor). This information was used to create a variable representing the network diversity of each participant. Following Cohen’s Social Network Index (Cohen et al. 1997; Ellwardt et al. 2015; Ali et al. 2018), network diversity was operationalized as the number of social roles represented in a person’s social network. With regard to the classification of social role categories, we used Ellwardt et al.’s (2015) adaptation of the index to typical social roles of older adults in the Netherlands, which we consider comparable to the German sociocultural context. The variables we defined were: spouse, child, child-in-law, sibling, sibling-in-law, parent, relative, close friend, acquaintance, neighbor, (former) colleague, voluntary organization, other. One point was assigned for each social role in the participants’ social network, multiple mentions of the same social role were counted only once. The sum score resulted in the *network diversity score*. Since information on social role could only be reported for up to eight network members, the maximum score was eight. A higher sum score indicated higher network diversity.

#### Control variables

We used age, sex, birth region (former East and West Germany), education, physical functioning, loneliness, marital status, employment status and community size as control variables. The rationale for the selection of control variables and explanations of their operationalization can be found in Online Resource 1.

#### Variables for sensitivity analysis

In order to test the robustness of the results based on the network diversity score, we conducted sensitivity analyses using different social role constellations. To this end, we created role groups for the network diversity score following categories from Litwin et al. (2020) and Litwin and Stoeckel (2014). The groups were (1) partner, (2) children, (3) other family members (e.g., siblings, grandchildren, parents), (4) friends and (5) others (e.g., neighbors, (ex-)colleagues). Respondents were coded with 1 (role group present) if one of these social roles was present in their social network, and coded with 0 if this was not the case. This was used to test for an association between the prevalence of each role group and SPA in the sensitivity analyses.

### Statistical analysis

Descriptive statistics comprise means and standard deviation for continuous measures as well as frequencies and percent for categorical measures. Bivariate linear associations were examined using Pearson correlations. We present the frequency of different social roles for different, equally sized age groups (40–49; 50–57; 58–66; 67–72; 73–85). Distributions were also analyzed by continuous age and did not differ.

Since this study is not interested in overall population frequencies or means but in associations, we did not consider the sampling weights of the DEAS. However, because the effects of selective non-response were unknown, selective non-response was examined using multiple logistic regression. This approach was chosen to correct for possible bias due to informative non-response using inverse probability weighting (Little et al. 2022). The complete procedure followed in order to generate the weights used for the analyses can be found in Online Resource 2. The weighting procedure corrected for selective non-response and approximately similar distributions of covariates were obtained as those observed in the DEAS 2008 sample (see Table 1).

Weighted multiple linear regression models adjusted for all covariates were used to model the association between network diversity and SPA. We did not include information on network size in the model specification due to its strong correlation with network diversity. Network diversity was entered into the models as a categorical variable since we did not assume there would be a common unit increase or decrease across all levels of the score; additionally, we examined the individual contributions of role groups in sensitivity analyses. In line with the literature (Peng and MacKenzie 2014), a network diversity score of 1 was selected as the reference category since this was the score most frequently observed compared to a score of 0. Likewise, the most frequently observed category was selected as the reference category for the categorical control variables. These are *male* for the variable *sex*, *medium education* for *education*, *retired* for *employment status*, *married/life partnership* for *marital status*, *Western Germany* for the variable *birth region* and the category *[5000–20,000) inhabitants* for the variable *community size*. In each model, we also tested for an interaction term of chronological age and network diversity. The term was retained in the model if information criteria (AIC; Burnham et al. 2004) and the likelihood ratio statistic (Harrell 2015) so suggested. In the same way as above, the interaction term of age and a network diversity score of 1 was selected as the reference category.

We applied multiple linear regression models to test for an association between different role groups and SPA in the sensitivity analyses and included chronological age and a single role group in each model. The models were applied to the weighted and the unweighted data set and tested both with and without an interaction term of chronological age and the respective role group. Results are presented as effect estimates and 95% confidence intervals. Except for likelihood ratio statistics, we do not present *p* values in line with recent methodological recommendations (Wasserstein et al. 2019; Amrhein et al. 2019) and discuss results based on their effect size and associated uncertainty.

## Results

### Descriptive statistics

Table 1 presents the sample characteristics of the study variables. The weighted sample comprised 6207 individuals. Respondents were on average 61.49 years old (SD = 11.97). 49.4 percent were female; 34.3 percent were highly educated. 70.2 percent were married or living in a partnership. The majority of respondents (46.6 percent) were retired, followed by 37.6 percent who were in part-time or full-time employment.

**Table 1:** Descriptive statistics of the study variables

Variable	DEAS 2008		
	Interview only	Interview and drop-off	Interview and drop-off weighted
*n*	6205	4238	6207
Age (mean (SD))	61.50 (12.10)	61.62 (11.84)	61.49 (11.97)
Sex (women) (%)	3072 (49.5)	2030 (47.9)	3068.2 (49.4)
*Employment status (%)*			
Part-time or full-time	2312 (37.3)	1562 (36.9)	2333.6 (37.6)
Early retirement	200 (3.2)	146 (3.4)	203.8 (3.3)
Unemployed	265 (4.3)	183 (4.3)	267.4 (4.3)
Other	168 (2.7)	103 (2.4)	173.9 (2.8)
Housewife, househusband	229 (3.7)	147 (3.5)	234.6 (3.8)
Marginal or secondary employment	98 (1.6)	61 (1.4)	99.3 (1.6)
Retired	2820 (45.4)	2036 (48.0)	2894.6 (46.6)
MISSING	113 (1.8)		
*Marital status (%)*			
Married / Life partnership	4352 (70.1)	3052 (72.0)	4356.4 (70.2)
Married (living separated)	84 (1.4)	56 (1.3)	84.0 (1.4)
Divorced	567 (9.1)	356 (8.4)	574.4 (9.3)
Widowed	741 (11.9)	489 (11.5)	736.8 (11.9)
Single	451 (7.3)	285 (6.7)	455.8 (7.3)
MISSING	10 (0.2)		
*Birth region (%)*			
Eastern Germany	2101 (33.9)	1533 (36.2)	2125.1 (34.2)
Western Germany	3507 (56.5)	2373 (56.0)	3497.7 (56.3)
Abroad	590 (9.5)	332 (7.8)	584.5 (9.4)
MISSING	7 (0.1)		
*Community size (%)*			
< 2000 inhabitants	783 (12.6)	574 (13.5)	785.3 (12.7)
[2000–5000) inhabitants	744 (12.0)	498 (11.8)	730.7 (11.8)
[5000–20,000) inhabitants	1743 (28.1)	1143 (27.0)	1740.5 (28.0)
[20,000–50,000) inhabitants	880 (14.2)	596 (14.1)	885.6 (14.3)
[50,000–100,000) inhabitants	518 (8.3)	341 (8.0)	520.1 (8.4)
[100,000–500,000) inhabitants	903 (14.6)	642 (15.1)	905.3 (14.6)
[> 500,000 inhabitants)	633 (10.2)	444 (10.5)	639.8 (10.3)
MISSING	1 (0.0)		
*Education (%)*			
medium (ISCED 3–4)	3374 (54.4)	2320 (54.7)	3409.4 (54.9)
low (ISCED 0–2)	716 (11.5)	383 (9.0)	669.7 (10.8)
high (ISCED 5–6)	2112 (34.0)	1535 (36.2)	2128.3 (34.3)
MISSING	3 (0.0)		
Physical functioning (mean (SD))	83.71 (23.22)	84.90 (21.84)	83.66 (23.26)
MISSING	28 (0.5)		
Network size (mean (SD))	4.30 (2.85)	4.43 (2.82)	4.32 (2.80)
MISSING	0 (0.0)		
Network diversity (mean (SD))	2.62 (1.50)	2.69 (1.48)	2.64 (1.48)
MISSING	29 (0.5)		
Loneliness (mean (SD))		1.75 (0.56)	1.76 (0.57)
SPA ongoing development (mean (SD))		2.89 (0.62)	2.88 (0.63)
SPA social losses (mean (SD))		1.87 (0.60)	1.88 (0.61)
SPA physical losses (mean (SD))		2.80 (0.56)	2.81 (0.57)

*SPA* self-perceptions of aging, Data source: German Ageing Survey (DEAS 2008)

**Fig. 1:**
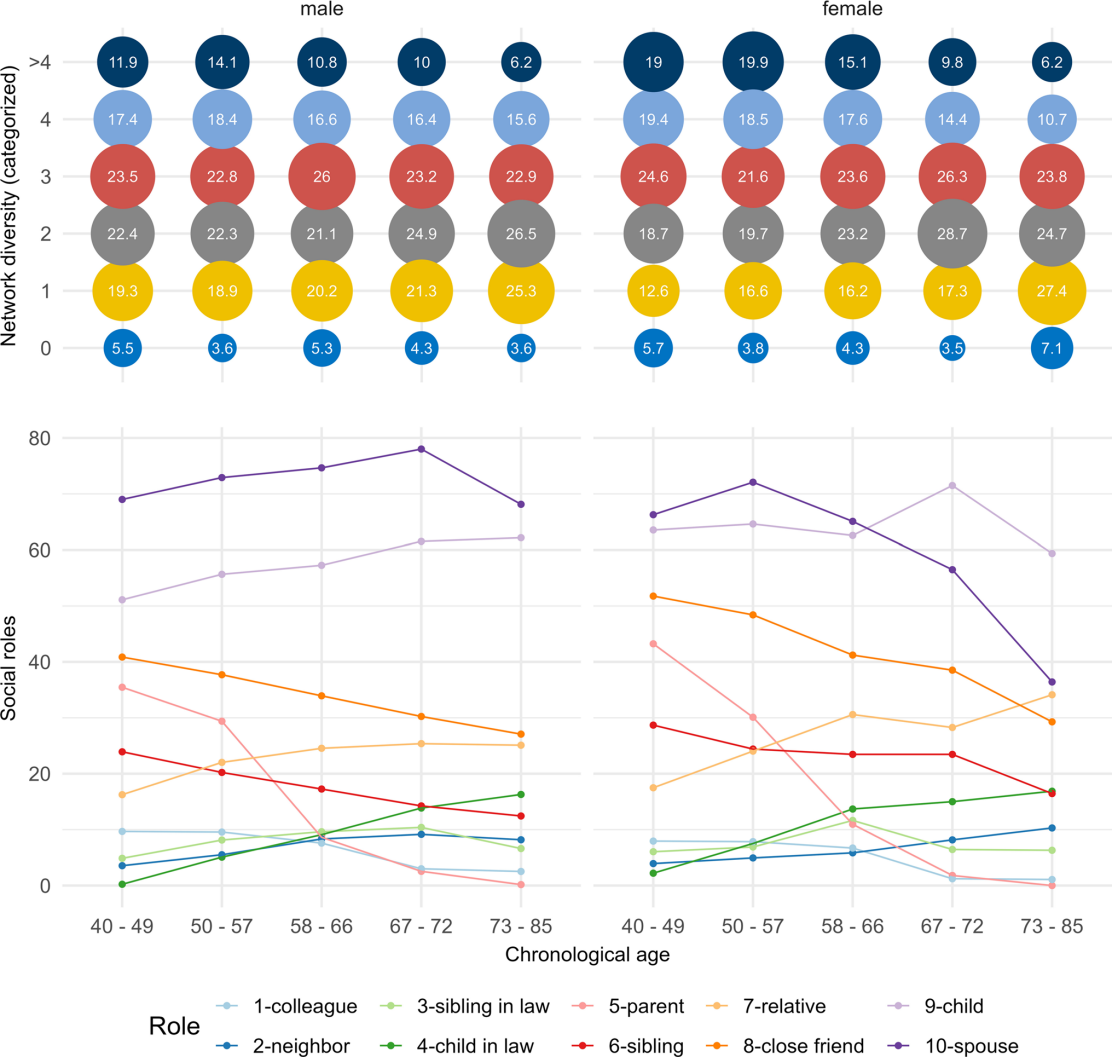
Frequency of social roles and of the categorized network diversity score in percent by sex along age groups (40–49, 50–57, 58–66, 67–72, 73–85). The figure depicts the frequency of different social roles and of the categorized network diversity score for different, equally sized age groups (40–49; 50–57; 58–66; 67–72; 73–85). Distributions were also analyzed by continuous age and did not differ; Data source: German Ageing Survey (DEAS 2008)

The average network size was 4.32 ties (SD = 2.80) and the average network diversity score amounted to 2.64 (SD = 1.48) (Table 1). The most frequently mentioned roles were spouse (65.6%), child (60.5%) and close friend (37.7%). Distribution of social roles differed between male and female participants as well as between age groups (Fig. 1). On average, respondents associated their own aging slightly more with positive than with negative SPA. This was indicated by higher means for the positive SPA domain of ongoing development (*M* = 2.88; SD = 0.63) than for the negative SPA domains of social losses (*M* = 1.88; SD = 0.61) and physical losses (*M* = 2.81; SD = 0.57) (see Table 1). Results from bivariate correlation analyses can be found in Online Resource 3.

### Social network diversity and SPA

**Fig. 2:**
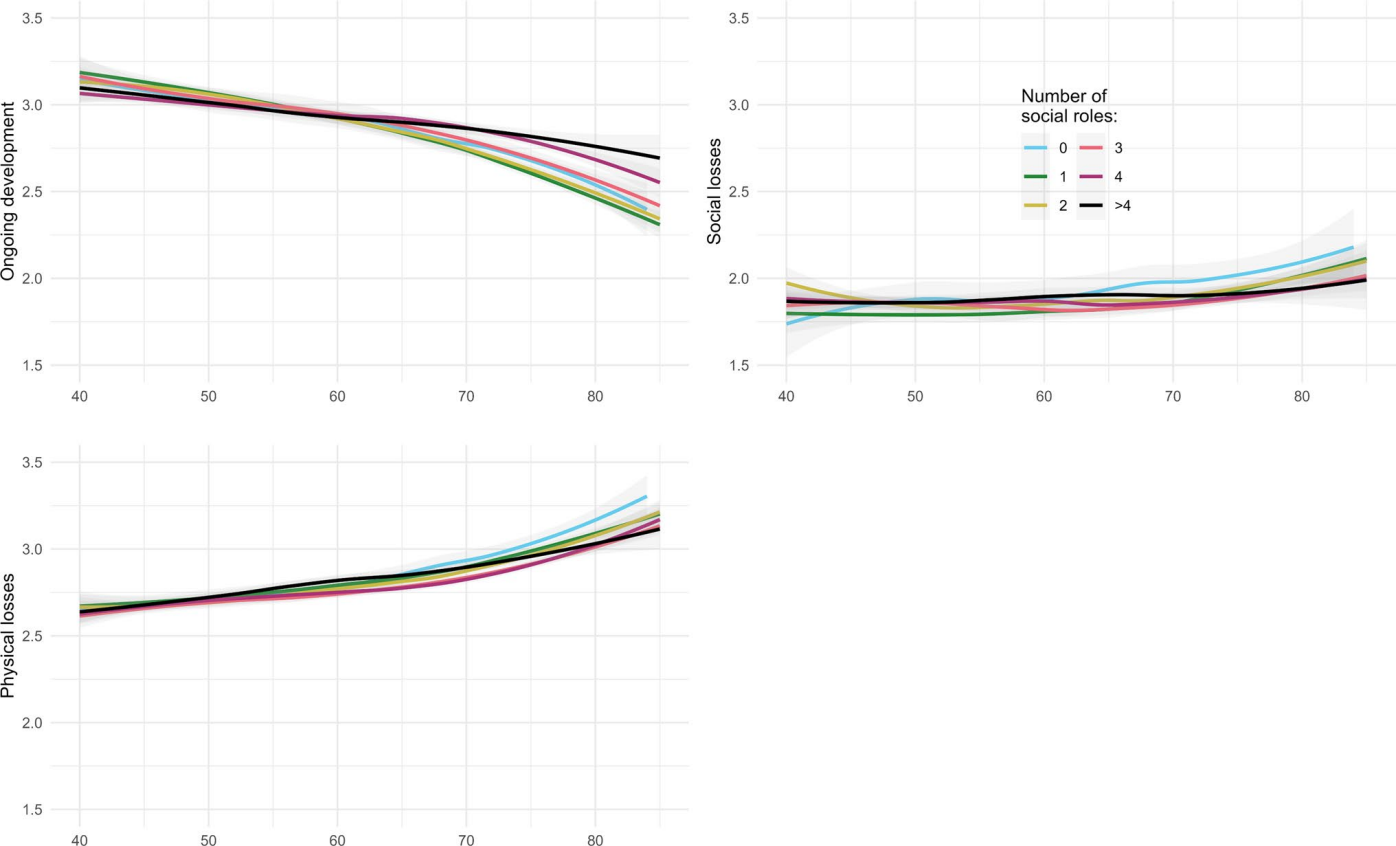
Mean values of each SPA domain by age and by network diversity score (number of social roles). *SPA* self-perceptions of aging, Data source: German Ageing Survey (DEAS 2008)

The initial regression model showed no relationship between network diversity and SPA ongoing development. In weighted multiple linear regression, we compared model 1 (without interaction term) with model 2 (including interaction). As indicated by information criteria (AIC_model 1_ = 7036; AIC_model 2_ = 7027) and the likelihood ratio statistic (*p* = 0.003), we then proceeded with model 2 (for linear contrasts in number of social roles for different age groups, see results from ANOVA in Online Resource 4). This model revealed a positive association between network diversity and SPA ongoing development moderated by chronological age (*β* (Network diversity > 4) = 0.01, 95% CI [− 0.05; 0.08]; *β* (Age × Network diversity > 4) = 0.10, 95% CI [0.04; 0.17]; Table 2). Less network diversity was associated with less positive SPA ongoing development in older age (Fig. 2).

**Table 2:** Regression model of SPA related to ongoing development

Variable	Model 1: without interaction (AIC: 7036)			Model 2: with interaction (AIC: 7027; LR: *p* = 0.003)		
	Estimate	*p* value	CI	Estimate	*p* value	CI
(Intercept)	2.75	0	[2.64; 2.87]	2.76	0	[2.65; 2.87]
Age	− 0.08	0	[− 0.11; − 0.05]	− 0.12	0	[− 0.17; − 0.08]
Sex (female)	0.02	0.175	[− 0.01; 0.06]	0.03	0.134	[− 0.01; 0.06]
*Education (medium (ISCED 3–4)) (ref.)*						
Education (low (ISCED 0–2))	− 0.1	0.001	[− 0.16; − 0.04]	− 0.1	0.001	[− 0.16; − 0.05]
Education (high (ISCED 5–6))	0.12	0	[0.08; 0.15]	0.12	0	[0.08; 0.15]
*Employment status (retired) (ref.)*						
Employment status (part-time or full-time)	0.1	0.004	[0.03; 0.16]	0.1	0.003	[0.03; 0.17]
Employment status (early retirement)	0.02	0.7	[− 0.08; 0.12]	0.02	0.727	[− 0.09; 0.12]
Employment status (unemployed)	0.03	0.56	[− 0.07; 0.13]	0.03	0.585	[− 0.07; 0.13]
Employment status (other)	0.21	0	[0.1; 0.32]	0.21	0	[0.1; 0.32]
Employment status (housewife, househusband)	0.12	0.02	[0.02; 0.22]	0.12	0.02	[0.02; 0.22]
Employment status (marginal or secondary employment)	− 0.13	0.081	[− 0.27; 0.02]	− 0.13	0.082	[− 0.27; 0.02]
*Marital status* *(married /* *life partnership)* *(ref.)*						
Marital status (married (living separated))	− 0.07	0.335	[− 0.22; 0.07]	− 0.08	0.286	[− 0.22; 0.07]
Marital status (divorced)	0.06	0.056	[0; 0.12]	0.05	0.078	[− 0.01; 0.11]
Marital status (widowed)	0.01	0.76	[− 0.05; 0.07]	0.01	0.606	[− 0.04; 0.07]
Marital status (single)	− 0.08	0.014	[− 0.15; − 0.02]	− 0.09	0.008	[− 0.16; − 0.02]
*Birth region (Western Germany) (ref.)*						
Birth region (Eastern Germany)	− 0.14	0	[− 0.18; − 0.11]	− 0.14	0	[− 0.18; − 0.11]
Birth region (abroad)	− 0.14	0	[− 0.2; − 0.08]	− 0.13	0	[− 0.19; − 0.07]
*Community size [5.000–20.000) inhabitants (ref.)*						
Community size < 2000 inhabitants	− 0.01	0.684	[− 0.07; 0.05]	− 0.01	0.684	[− 0.07; 0.05]
Community size [2000–5000) inhabitants	− 0.08	0.005	[− 0.14; − 0.02]	− 0.08	0.006	[− 0.14; − 0.02]
Community size [20,000–50,000) inhabitants	− 0.01	0.765	[− 0.06; 0.05]	− 0.01	0.792	[− 0.06; 0.05]
Community size [50,000–100,000) inhabitants	0.05	0.159	[− 0.02; 0.11]	0.05	0.156	[− 0.02; 0.11]
Community size [100,000–500,000) inhabitants	0.04	0.155	[− 0.01; 0.09]	0.04	0.157	[− 0.01; 0.09]
Community size [> 500,000 inhabitants)	0	0.926	[− 0.06; 0.06]	0	0.877	[− 0.06; 0.07]
*Network diversity = 1 (ref.)*						
Network diversity = 0	0.04	0.344	[− 0.04; 0.12]	0.03	0.424	[− 0.05; 0.12]
Network diversity = 2	0	0.886	[− 0.05; 0.05]	0	0.951	[− 0.05; 0.05]
Network diversity = 3	− 0.01	0.709	[− 0.06; 0.04]	− 0.02	0.525	[− 0.07; 0.03]
Network diversity = 4	0	0.983	[− 0.06; 0.06]	0	0.993	[− 0.06; 0.06]
Network diversity > 4	0	0.993	[− 0.06; 0.06]	0.01	0.687	[− 0.05; 0.08]
Loneliness	− 0.24	0	[− 0.27; − 0.21]	− 0.24	0	[− 0.27; − 0.21]
Physical functioning	0.06	0	[0.05; 0.07]	0.06	0	[0.05; 0.07]
*Age × Network diversity = 1 (ref.)*						
Age × Network diversity = 0				0.07	0.095	[− 0.01; 0.14]
Age × Network diversity = 2				0.02	0.423	[− 0.03; 0.07]
Age × Network diversity = 3				0.03	0.285	[− 0.02; 0.08]
Age × Network diversity = 4				0.09	0.001	[0.04; 0.15]
Age × Network diversity > 4				0.1	0.002	[0.04; 0.17]

*SPA* self-perceptions of aging, *AIC* Akaike information criterion, *LR* likelihood ratio statistic, Data source: German Ageing Survey (DEAS 2008)

For SPA social losses, the regression model displayed a small, significant association between network diversity and this SPA domain. However, this relationship was found to be positive (*β* (Network diversity > 4) = 0.10, 95% CI [0.04; 0.15]; Table 3). Additionally, information criteria (AIC_model 1_ = 6487; AIC_model 2_ = 6486) and the likelihood ratio statistic (*p* = 0.048) indicated the relevance of an interaction term with age. The final model, including the interaction term, shows that at older ages higher network diversity is associated with more negative SPA social losses (β (Network diversity > 4) = 0.10, 95% CI [0.03; 0.16]; *β* (Age × Network diversity > 4) = −0.06, 95% CI [− 0.12; 0]; Table 3).

**Table 3:** Regression model of SPA related to social losses

Variable	Model 1: without interaction (AIC: 6487)			Model 2: with interaction (AIC: 6486; LR: *p* = 0.048)		
	Estimate	*p* value	CI	Estimate	*p* value	CI
(Intercept)	1.27	0	[1.17; 1.38]	1.27	0	[1.16; 1.37]
Age	0.01	0.451	[− 0.02; 0.04]	0.05	0.019	[0.01; 0.09]
Sex (female)	− 0.05	0.008	[− 0.08; − 0.01]	− 0.05	0.006	[− 0.08; − 0.01]
*Education (medium (ISCED 3–4)) (ref.)*						
Education (low (ISCED 0–2))	0.09	0.001	[0.04; 0.15]	0.09	0.001	[0.04; 0.15]
Education (high (ISCED 5–6))	− 0.07	0	[− 0.1; − 0.03]	− 0.07	0	[− 0.1; − 0.03]
*Employment status (retired) (ref.)*						
Employment status (part-time or full-time)	− 0.01	0.774	[− 0.07; 0.05]	− 0.01	0.737	[− 0.07; 0.05]
Employment status (early retirement)	0.12	0.016	[0.02; 0.22]	0.12	0.014	[0.03; 0.22]
Employment status (unemployed)	0.1	0.035	[0.01; 0.19]	0.1	0.033	[0.01; 0.19]
Employment status (other)	0.13	0.01	[0.03; 0.23]	0.13	0.012	[0.03; 0.23]
Employment status (housewife, househusband)	− 0.01	0.844	[− 0.1; 0.08]	− 0.01	0.857	[− 0.1; 0.09]
Employment status (marginal or secondary employment)	0.06	0.382	[− 0.08; 0.2]	0.06	0.366	[− 0.07; 0.2]
*Marital status (married / life partnership) (ref.)*						
Marital status (married (living separated))	0.08	0.246	[− 0.06; 0.22]	0.08	0.265	[− 0.06; 0.21]
Marital status (divorced)	0.06	0.033	[0; 0.12]	0.06	0.028	[0.01; 0.12]
Marital status (widowed)	0.15	0	[0.1; 0.2]	0.15	0	[0.1; 0.2]
Marital status (single)	0.12	0	[0.06; 0.19]	0.12	0	[0.06; 0.19]
*Birth region (Western Germany) (ref.)*						
Birth region (Eastern Germany)	0.03	0.071	[0; 0.07]	0.03	0.072	[0; 0.07]
Birth region (abroad)	0.06	0.052	[0; 0.11]	0.05	0.065	[0; 0.11]
*Community size [5000–20,000) inhabitants (ref.)*						
Community size < 2000 inhabitants	− 0.06	0.034	[− 0.11; 0]	− 0.06	0.039	[− 0.11; 0]
Community size [2000–5000) inhabitants	0.02	0.47	[− 0.03; 0.07]	0.02	0.469	[− 0.03; 0.07]
Community size [20,000–50,000) inhabitants	− 0.01	0.582	[− 0.07; 0.04]	− 0.01	0.629	[− 0.06; 0.04]
Community size [50,000–100,000) inhabitants	0	0.996	[− 0.06; 0.06]	0	0.978	[− 0.06; 0.06]
Community size [100,000–500,000) inhabitants	− 0.02	0.544	[− 0.07; 0.03]	− 0.02	0.554	[− 0.07; 0.04]
Community size [> 500,000 inhabitants)	− 0.04	0.2	[− 0.09; 0.02]	− 0.04	0.197	[− 0.1; 0.02]
*Network diversity = 1 (ref.)*						
Network diversity = 0	0.02	0.706	[− 0.06; 0.09]	0.02	0.595	[− 0.06; 0.1]
Network diversity = 2	0.05	0.032	[0; 0.1]	0.06	0.019	[0.01; 0.1]
Network diversity = 3	0.05	0.033	[0; 0.1]	0.06	0.017	[0.01; 0.11]
Network diversity = 4	0.08	0.002	[0.03; 0.14]	0.09	0.001	[0.03; 0.14]
Network diversity > 4	0.1	0.002	[0.04; 0.15]	0.1	0.002	[0.03; 0.16]
Loneliness	0.47	0	[0.44; 0.5]	0.47	0	[0.44; 0.5]
Physical functioning	− 0.03	0	[− 0.04; − 0.03]	− 0.03	0	[− 0.04; − 0.03]
*Age × Network diversity = 1 (ref.)*						
Age × Network diversity = 0				− 0.08	0.027	[− 0.16; − 0.01]
Age × Network diversity = 2				− 0.02	0.378	[− 0.07; 0.03]
Age × Network diversity = 3				− 0.06	0.012	[− 0.11; − 0.01]
Age × Network diversity = 4				− 0.06	0.035	[− 0.11; 0]
Age × Network diversity > 4				− 0.06	0.049	[− 0.12; 0]

*SPA* self-perceptions of aging, *AIC* Akaike information criterion, *LR* likelihood ratio statistic, Data source: German Ageing Survey (DEAS 2008)

Finally, network diversity was not associated with SPA physical losses (*β* (Network diversity > 4) = 0.03, 95% CI [− 0.03; 0.09]; Table 4). Furthermore, according to information criteria (AIC_model 1_ = 6477; AIC_model 2_ = 6484) and the likelihood ratio statistic (*p* = 0.764), the interaction term did not contribute to model improvement.

**Table 4:** Regression model of SPA related to physical losses

Variable	Model 1: without interaction (AIC: 6477)			Model 2: with interaction (AIC: 6484; LR: *p* = 0.764)		
	Estimate	*p* value	CI	Estimate	*p* value	CI
(Intercept)	3.31	0	[3.2; 3.41]	3.3	0	[3.2; 3.41]
Age	0.05	0.001	[0.02; 0.08]	0.07	0.001	[0.03; 0.11]
Sex (female)	− 0.03	0.103	[− 0.06; 0.01]	− 0.03	0.095	[− 0.06; 0]
*Education (medium (ISCED 3–4)) (ref.)*						
Education (low (ISCED 0–2))	0.06	0.026	[0.01; 0.12]	0.06	0.024	[0.01; 0.12]
Education (high (ISCED 5–6))	− 0.01	0.721	[− 0.04; 0.03]	− 0.01	0.711	[− 0.04; 0.03]
*Employment status (retired) (ref.)*						
Employment status (part-time or full-time)	− 0.01	0.819	[− 0.07; 0.05]	− 0.01	0.782	[− 0.07; 0.05]
Employment status (early retirement)	0.03	0.579	[− 0.07; 0.12]	0.03	0.576	[− 0.07; 0.13]
Employment status (unemployed)	0.02	0.692	[− 0.07; 0.11]	0.02	0.678	[− 0.07; 0.11]
Employment status (other)	− 0.04	0.474	[− 0.14; 0.06]	− 0.04	0.452	[− 0.14; 0.06]
Employment status (housewife, househusband)	− 0.1	0.037	[− 0.19; − 0.01]	− 0.1	0.036	[− 0.2; − 0.01]
Employment status (marginal or secondary employment)	0.1	0.152	[− 0.04; 0.24]	0.1	0.15	[− 0.04; 0.24]
*Marital status (married / life partnership) (ref.)*						
Marital status (married (living separated))	0.1	0.139	[− 0.03; 0.24]	0.1	0.132	[− 0.03; 0.24]
Marital status (divorced)	− 0.01	0.815	[− 0.06; 0.05]	− 0.01	0.841	[− 0.06; 0.05]
Marital status (widowed)	0	0.859	[− 0.05; 0.06]	0	0.923	[− 0.05; 0.06]
Marital status (single)	0.06	0.085	[− 0.01; 0.12]	0.06	0.074	[− 0.01; 0.12]
*Birth region (Western Germany) (ref.)*						
Birth region (Eastern Germany)	0.06	0.002	[0.02; 0.09]	0.06	0.002	[0.02; 0.09]
Birth region (abroad)	0.06	0.057	[0; 0.11]	0.05	0.065	[0; 0.11]
*Community size [5000–20,000) inhabitants (ref.)*						
Community size < 2000 inhabitants	0.07	0.008	[0.02; 0.13]	0.07	0.008	[0.02; 0.13]
Community size [2000–5000) inhabitants	0.04	0.136	[− 0.01; 0.1]	0.04	0.14	[− 0.01; 0.1]
Community size [20,000–50,000) inhabitants	0.02	0.555	[− 0.04; 0.07]	0.02	0.549	[− 0.04; 0.07]
Community size [50,000–100,000) inhabitants	0.06	0.041	[0; 0.13]	0.06	0.041	[0; 0.13]
Community size [100,000–500,000) inhabitants	− 0.02	0.502	[− 0.07; 0.03]	− 0.02	0.491	[− 0.07; 0.03]
Community size [> 500,000 inhabitants)	− 0.06	0.06	[− 0.11; 0]	− 0.06	0.059	[− 0.11; 0]
*Network diversity = 1 (ref.)*						
Network diversity = 0	0.02	0.689	[− 0.06; 0.09]	0.02	0.625	[− 0.06; 0.1]
Network diversity = 2	− 0.02	0.499	[− 0.06; 0.03]	− 0.01	0.618	[− 0.06; 0.04]
Network diversity = 3	− 0.02	0.511	[− 0.06; 0.03]	− 0.01	0.615	[− 0.06; 0.04]
Network diversity = 4	− 0.01	0.827	[− 0.06; 0.05]	0	0.871	[− 0.06; 0.05]
Network diversity > 4	0.03	0.317	[− 0.03; 0.09]	0.03	0.345	[− 0.03; 0.09]
Loneliness	0.09	0	[0.06; 0.12]	0.09	0	[0.06; 0.12]
Physical functioning	− 0.08	0	[− 0.09; − 0.07]	− 0.08	0	[− 0.09; − 0.07]
*Age *×* Network diversity = 1 (ref.)*						
Age × Network diversity = 0				− 0.03	0.435	[− 0.1; 0.04]
Age × Network diversity = 2				− 0.03	0.26	[− 0.07; 0.02]
Age × Network diversity = 3				− 0.02	0.46	[− 0.06; 0.03]
Age × Network diversity = 4				− 0.04	0.178	[− 0.09; 0.02]
Age × Network diversity > 4				− 0.04	0.252	[− 0.1; 0.03]

*SPA* self-perceptions of aging, *AIC* Akaike information criterion, *LR* likelihood ratio statistic, Data source: German Ageing Survey (DEAS 2008)

### Sensitivity analysis

Regression models conducted as sensitivity analyses with different social role groups revealed that such groups had heterogeneous effects on SPA (see Online Resource 5). In the weighted data set, the prevalence of the group *partner* was negatively associated with SPA social losses (*β*_role group *partner*_ = − 0.194, 95% CI [− 0.232; − 0.155]), suggesting that individuals with a partner associated aging to a lesser extent with social losses. In contrast, a positive association was found between the prevalence of the group *others* and SPA social losses (*β*_role group *others*_ = 0.125, 95% CI [0.076; 0.174]; see Online Resource 5). Smaller but equally heterogeneous effects were found for SPA ongoing development in the weighted data set. Taking into account the interaction term of age and role group, the positive association between the group *friends* and SPA ongoing development increased with age (*β*_role group *friends*_ = 0.032, 95% CI [− 0.006; 0.069]; *β*_Age x role group *friends*_ = 0.04, 95% CI [− 0.008; 0.088]), while the positive relationship between the group *children* and SPA ongoing development decreased with age (β_role group *children*_ = 0.011, 95% CI [− 0.027; 0.048]; *β*_Age x role group *children*_ = − 0.01, 95% CI [− 0.047; 0.027]; Online Resource 5).

## Discussion

The purpose of this study was to examine the relationship between network diversity and three domains of SPA. We hypothesized that more diverse social networks would be related to more positive SPA related to ongoing development (H1), less negative SPA related to social losses (H2) and less negative SPA related to physical losses (H3). Furthermore, we assumed that this association would be moderated by chronological age, i.e., that the relationship would become more pronounced in older age (H4). With regard to SPA ongoing development, our study found support for both hypotheses H1 and H4: Considering an interaction between chronological age and network diversity revealed that at higher ages, older adults with more diverse social networks had more positive perceptions of ongoing development than older adults with less diverse social networks. Regarding SPA social losses, our results contradict H2 in that we found a small positive (rather than negative) association between network diversity and SPA social losses. In line with H4, we found that chronological age did indeed have a moderating effect, allowing us to conclude for SPA social losses that at older ages, individuals with more diverse networks associate aging more strongly with social losses than those individuals with less diverse networks. Furthermore, in contradiction to both H3 and H4, we found no association between network diversity and SPA physical losses and no indication that chronological age is of relevance to the relationship.

Several aspects of our results merit comment. Overall, in line with previous findings (Beyer et al. 2017), SPA were found to be less positive and more negative in older compared to younger ages. However, our results suggest that network diversity exerts a cushioning effect on the decrease in the perception of ongoing development across the age groups. This finding is in line with the assumptions of the DIRe model that older adults can benefit from network diversity (Huxhold et al. 2022). However, the question of whether diverse social networks compensate for age-related changes, as suggested in the DIRe model (Huxhold et al. 2022), could not be examined in the present, cross-sectional study. The results of qualitative studies indicate that the loss of social roles that comes along with some age-related events such as retirement may challenge an individual’s sense of purpose in life (Hobbis et al. 2011; Jones et al. 2010). Maintaining purpose in life by performing various social roles and exploiting the stimulating potential and social opportunities offered by a diverse social network might contribute to upholding the perception of ongoing development in older age.

However, our results suggest that network diversity is also slightly positively associated with negative SPA, that is, SPA social losses. One possible explanation for this is related to the ambiguity of the concept and the operationalization of SPA. The item stem of the AgeCog scales “Ageing means to me…” permits both retrospective and prospective associations with one's own aging. Therefore, it is possible that the relationship between network diversity and SPA social losses reflects anticipation on the part of those individuals with diverse networks that they might lose social roles in the future. The finding that network diversity did not play a role in SPA physical losses suggests that diverse social networks do not offer any considerable advantages for age-related perceptions of physical health. Past research indicates that the support essential to buffering against stress is provided by few close relationships (Cohen and Wills 1985) and that instrumental support to older adults is mostly provided by children (Seeman and Berkman 1988). This suggests that the fulfillment of essential needs regarding physical health in old age is assured by a network consisting of just a few, similar social roles, and that network diversity is not of relevance in this domain.

The observed effects, however, might also be explained in the reverse direction. According to the DIRe model (Huxhold et al. 2022), social networks, individual and contextual factors are interrelated. Experiencing social losses such as the death of a partner could lead to the loss of further network contacts and thus to reduced network diversity. SPA related to ongoing development could also motivate older adults to invest more into different types of social roles (Huxhold et al. 2022). This would tie in with the relationship between positive SPA and new friendships in older adults shown in a study by Menkin et al. (2017).

The sensitivity analyses based on different social role constellations complement our findings on the association between network diversity and SPA. Heterogeneous effects for the different role groups suggest that the different roles have a distinct relevance for SPA, which in turn supports the association revealed by our study between network diversity and SPA ongoing development and SPA social losses.

The present results extend existing knowledge on the relationship between structural network characteristics and SPA, which has been based on network size and age integration. The results of our study show that network diversity plays a role in both positive and negative SPA. The results provide partial and tentative support for the assumptions of the DIRe model, although they only suggest a beneficial role for network diversity in age-related perceptions of ongoing development and can clarify neither the direction of effect nor the mechanisms behind it. In addition, the present study contributes to an understanding of which social structures play a role in the aging experience of older adults. This knowledge could ultimately prove important for recommending measures to maintain or create specific social opportunity structures, including multigenerational homes, programs and services based on interests rather than age, or the promotion of professional and post-professional engagement to maintain certain social roles, just to give some examples. As SPA are considered to play an important role in successful aging (Sabatini et al. 2024), our findings thus represent a useful addition to existing studies by shedding light on the role of network diversity in SPA in later life.

## Limitations and directions for future research

We acknowledge that this study has several limitations. One weakness concerns the measurement of social networks applied in the DEAS questionnaire. This instrument is based on the participants’ self-assessment of people “whom they considered important and with whom they had regular contact.” Both the item wording and the dependence on the respondents' memory could entail peripheral contacts from everyday life not being recalled or not being perceived as important enough to report. A useful approach to recording the diverse social contacts in older adults’ everyday lives more reliably is the Ecological Momentary Assessment, in which respondents provide information about their social encounters at regular intervals several times a day (Fingerman et al. 2020). This could provide a more accurate picture of how diverse the networks of older people are, and reveal different results on the relationship with SPA.

Moreover, the network diversity score which we calculated entails a simplification of network diversity. Grouping the social roles into an index cannot reflect differences between role constellations with the same index value. To address this deficit, we conducted sensitivity analyses based on specific social role groups. Results indicate that associations with SPA differ from role group to role group. Statistical procedures for identifying network types and their distinctive associations with SPA were not feasible within the scope of this study due to the large number of role combinations and the additional factor of variation with age. However, in view of the differing effects of different role groups, analyses of this nature would seem to offer a promising approach for future studies. Furthermore, adding qualitative properties of relationships to future investigations could contribute to a more differentiated understanding of specific roles.

Due to its sampling criteria, the DEAS only provides data on older adults living in private households in the community (Klaus et al. 2017). The present findings can thus not be transferred to older adults living in institutions, in which network diversity would appear less likely.

Lastly, conclusions that can be drawn from our findings are limited by the fact that the results are based on cross-sectional analyses. Thus, while the study provides initial evidence on the association between network diversity and SPA, further longitudinal investigations are required to better understand the direction of the relationship.

## Conclusion

Our results indicate that the extent of network diversity is associated with how individuals perceive their own aging regarding ongoing development and social losses. For both SPA domains, the findings further suggest that network diversity gains in importance among older age groups. Future studies should longitudinally examine the interrelationship between network diversity and SPA, and the role of age therein.

### Supplementary Information

Below is the link to the electronic supplementary material.

Supplementary Material 1.

Supplementary Material 2.

Supplementary Material 3.

Supplementary Material 4.

Supplementary Material 5.

## Data Availability

The data set analyzed in the present study is available free of charge to scientific researchers for non-profitable purposes on request to the German Research Data Centre of the German Centre of Gerontology (DZA).
