# Supplementary material for: The role of social network diversity in self-perceptions of aging in later life
Source: Eur J Ageing. 2024 Jun 26;21(1):20. doi: 10.1007/s10433-024-00815-z (PMC11208383; doi:10.1007/s10433-024-00815-z)
Supplement: Supplementary file 1 — Supplementary Material 1. [file 10433_2024_815_MOESM1_ESM.pdf]

The role of social network diversity in self-perceptions of aging in later life

European Journal of Ageing

Frauke Meyer-Wyk[1,2], Susanne Wurm[1]

[1]Institute for Community Medicine, Department for Prevention Research and Social Medicine, University Medicine, Greifswald, Germany

[2]European Commission, Joint Research Centre (JRC), Ispra, Italy

Corresponding author: Frauke Meyer-Wyk, frauke.meyer-wyk@med.uni-greifswald.de

Online Resource 1: Control variables

We used age, sex, birth region (former East and West Germany), education, physical functioning, loneliness, marital status, employment status and community size as control variables. Age and region have been found to be related to SPA (Beyer et al. 2017; Wurm and Huxhold 2012). Age and sex were related to size and composition of social networks in several studies (e.g. Wrzus et al. 2013; Schwartz and Litwin 2018). As the level of education has been shown to be related to SPA (Wurm and Huxhold 2012), we included education as a control variable. We also used this as a proxy for socioeconomic status, as income appeared less suitable due to high non-response rates in many German studies. The level of education was assessed according to the International Standard Classification of Education and subsequently summarized (ISCED: low (without formal vocational qualification), medium (with completed vocational training and/or high school Diploma) and high education (with completed professional development training and/or with completed university studies)). Due to previous findings in support of a relation between physical functioning and SPA (Sargent-Cox et al. 2012), we also controlled for physical functioning according to the subscale of the SF-36 Health Survey (Bullinger and Kirchberger 1998). Participants evaluated their limitation in ten daily activities on a scale from 1 (yes, limited a lot) to 3 (no, not limited at all). The sum of these items was transformed into a scale ranging from 1 to 100. A higher value indicated better physical functioning. Additionally, we included loneliness to control for confounding, as previous studies pointed to a relation of loneliness and SPA (Diehl et al. 2021) as well as loneliness and social isolation (Shankar et al. 2011). Loneliness was measured with a modified version of the De Jong Gierveld loneliness scale (De Jong Gierveld and van Tilburg 2006). The six items of the scale (e.g., “I miss having people who I feel comfortable with”) could be rated from 1 (Strongly agree) to 4 (Strongly disagree). The mean value of the six items accounted for the total score. A higher score indicated a higher level of loneliness. Furthermore, we controlled for marital status and employment status as we considered associations with network properties likely for both variables (such as network differences

between widowed and married or retired and employed individuals). The variable employment status was generated from the variables "current employment status" (DEAS variable x101) and "receipt of old-age pension/retirement benefits from previous job" (DEAS variable x100). Marital status is based on the query of respondents' civil status (DEAS variable x200). To control for confounding, we also included community size, since rural areas are likely to have different network structures than urban areas due to a higher proportion of older adults in rural areas (Henger and Oberst 2019). The variable community size contains the categorized population of the municipality of respondents' residence as of 31.12.2005 (DEAS variable polgk_08).

References

Beyer AK, Wurm S, Wolff JK (2017) Älter werden–Gewinn oder Verlust? Individuelle Altersbilder und Altersdiskriminierung. In: Mahne K, Wolff J, Simonson J, Tesch-Römer C (eds) Altern im Wandel. Springer Fachmedien, Wiesbaden, pp 329-343. https://doi.org/10.1007/978-3-658-12502-8_22

Bullinger M, Kirchberger I (1998) Der SF-36 Fragebogen zum Gesundheitszustand. Hogrefe Verlag, Göttingen

De Jong Gierveld J, Van Tilburg T (2006) A 6-item scale for overall, emotional, and social loneliness: Confirmatory tests on survey data. Res Aging 28:582-598. http://doi.org/10.1177/0164027506289723

Diehl M, Wettstein M, Spuling SM, Wurm S (2021) Age-related change in self-perceptions of aging: Longitudinal trajectories and predictors of change. Psychol Aging 36:344-359. https://dx.doi.org/10.1037/pag0000585

Henger R, Oberst C (2019) Alterung der Gesellschaft im Stadt-Land Vergleich. IW-Kurzbericht 16. Institut der deutschen Wirtschaft. http://hdl.handle.net/10419/195079

Sargent-Cox KA, Anstey KJ, Luszcz MA (2012) The relationship between change in self-perceptions of aging and physical functioning in older adults. Psychol Aging 27:750–760. https://doi.org/10.1037/a0027578

Schwartz E, Litwin H (2018) Social network changes among older Europeans: the role of gender. Eur J Ageing 15:359–367. https://doi.org/10.1007/s10433-017-0454-z

Shankar A, McMunn A, Banks J, Steptoe A (2011) Loneliness, social isolation, and behavioral and biological health indicators in older adults. Health Psychol 30:377-385. https://doi.org/10.1037/a0022826

Wrzus C, Hänel M, Wagner J, Neyer FJ (2013) Social network changes and life events across the life span: a meta-analysis. Psychol Bull 139:53-80. https://doi.org/10.1037/a0028601

Wurm S, Huxhold O (2012) Sozialer Wandel und individuelle Entwicklung von Altersbildern. In: Nachname Vorname (ed) Individuelle und kulturelle Altersbilder. VS Verlag für Sozialwissenschaften, Wiesbaden, pp 27-69
