# Supplementary material for: The role of social network diversity in self-perceptions of aging in later life
Source: Eur J Ageing. 2024 Jun 26;21(1):20. doi: 10.1007/s10433-024-00815-z (PMC11208383; doi:10.1007/s10433-024-00815-z)
Supplement: Supplementary file 2 — Supplementary Material 2. [file 10433_2024_815_MOESM2_ESM.pdf]

The role of social network diversity in self-perceptions of aging in later life

European Journal of Ageing

Frauke Meyer-Wyk[1,2], Susanne Wurm[1]

[1]Institute for Community Medicine, Department for Prevention Research and Social Medicine, University Medicine, Greifswald, Germany

[2]European Commission, Joint Research Centre (JRC), Ispra, Italy

Corresponding author: Frauke Meyer-Wyk, frauke.meyer-wyk@med.uni-greifswald.de

Online Resource 2: Weighting procedure

Since the present study is not interested in overall population frequencies or means, but in associations, we did not consider the sampling weights of the DEAS. However, because the effects of selective non-response were unknown, selective non-response in the 2008 panel of the DEAS was examined using multiple logistic regression. This approach was chosen to correct for possible bias due to informative non-response using inverse probability weighting (Little et al. 2022). First, we examined association of covariates with missing data in the *interview*. Therefore, we created a binary variable being 1 if the participant had missing values in one of the following covariates: employment status, marital status, birth region, community size, education, physical functioning, and network size. If none of these covariates had missing values, the binary variable has been coded to 0. The logistic regression model in this step comprised only age and sex as covariates. Missing values were observed for n=180 observations (29%). In a second step we proceeded identically, but now the occurrence of missing values in the paper-and-pencil questionnaire ('drop-off'), which contained the scales on SPA and loneliness, was coded binary (1 = missing values observed, 0 = no missing values). The multiple logistic regression model of this step comprised: age, sex, employment status, birth region, marital status, community size, education, physical functioning, and network size. Missing values were observed for n = 1,787 observations (28.8%). Probability of missing values was related to birth region (OR (abroad) = 1,78, 95 % CI [1.45; 2.19]), marital status (OR (divorced) = 1,41, 95 % CI [1.16; 1.70]) and education (OR (low (ISCED 0-2)) = 1,33, 95 % CI [1.10; 1.61]).

From logistic regression models the predicted probabilities for missing data of each participant were used to calculate inverse probability weights: $w_{1i} = \frac{1}{1-\widehat{p_{1i}}}$ and $w_{2i} = \frac{1}{1-\widehat{p_{2i}}}$. w_{1i} corresponds to the weight for missing values in the interview, w_{2i} for missing values in the paper-and-pencil questionnaire. The weight for non-response has been calculated as the product ($w_{1i} * w_{2i}$). Assessment of resulting weights showed no presence of extreme values

(min: 1.17, median: 1.53, mean: 2.08, max: 7.63). The weighting procedure corrected for selective non-response and approximately similar distributions of covariates were obtained as observed in 6,205 participants of the 2008 panel.

References

Little RJ, Carpenter JR, Lee KJ (2022) A comparison of three popular methods for handling missing data: complete-case analysis, inverse probability weighting, and multiple imputation. Sociol Methods Res:1-31. https://doi.org/10.1177/00491241221113873
