# Supplementary material for: The role of social network diversity in self-perceptions of aging in later life
Source: Eur J Ageing. 2024 Jun 26;21(1):20. doi: 10.1007/s10433-024-00815-z (PMC11208383; doi:10.1007/s10433-024-00815-z)
Supplement: Supplementary file 3 — Supplementary Material 3. [file 10433_2024_815_MOESM3_ESM.pdf]

The role of social network diversity in self-perceptions of aging in later life

European Journal of Ageing

Frauke Meyer-Wyk[1,2], Susanne Wurm[1]

[1]Institute for Community Medicine, Department for Prevention Research and Social Medicine, University Medicine, Greifswald, Germany

[2]European Commission, Joint Research Centre (JRC), Ispra, Italy

Corresponding author: Frauke Meyer-Wyk, frauke.meyer-wyk@med.uni-greifswald.de

Online Resource 3: Pearson correlations of the main study variables

Bivariate correlation coefficients among the study variables are presented in Figure 1. Network diversity was highly correlated with network size (*r* = 0.82), but did not display notable relations with other variables. Individuals who had higher perceptions of ongoing development were likely to be younger (*r* = -0.27), to have better physical functioning (*r* = 0.34) and lower levels of loneliness (*r* = -0.23). Higher perceptions of social losses and physical losses were inversely correlated with these variables, in particular SPA social losses and loneliness (*r* = 0.48) as well as SPA physical losses and physical functioning (*r* = -0.38).

Fig. 1 Pearson correlations of the main study variables

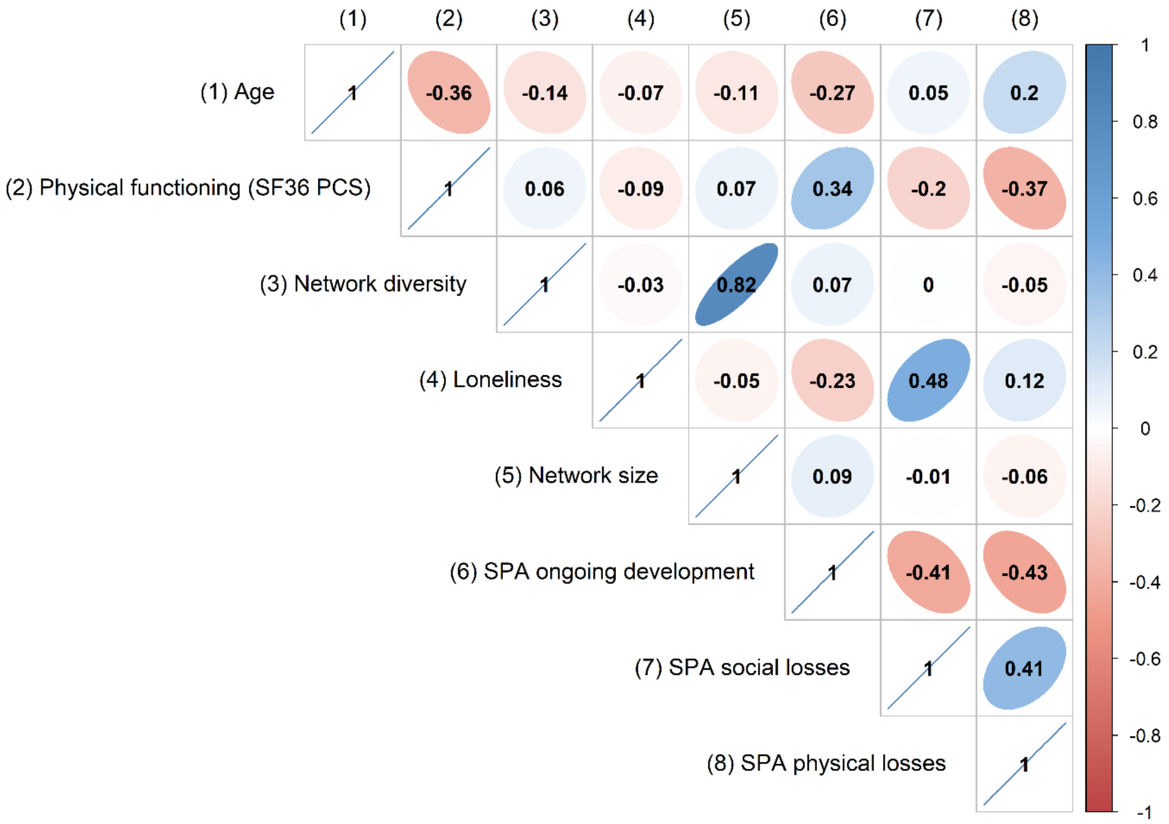

SF36 PCS short form 36 physical component score, *SPA* self-perceptions of aging, Data source: German Ageing Survey (DEAS 2008)
